# Are Emergency Medicine Provider Characteristics Associated With Diagnostic Imaging for Low Back Pain?

**DOI:** 10.7759/cureus.13628

**Published:** 2021-03-01

**Authors:** Jonathan Doucett, Jill Hayden, Kirk D Magee, Rachel Ogilvie

**Affiliations:** 1 Emergency Medicine, Dalhousie University, Halifax, CAN; 2 Research, Dalhousie University, Halifax, CAN

**Keywords:** diagnostic imaging, mechanical back pain, non-specific low back pain, emergency department, physician characteristics, low back pain

## Abstract

Background

Low back pain (LBP) is one of the most common reasons for presentation to the emergency department (ED). LBP is most commonly non-specific or mechanical in nature yet can be debilitating. Diagnostic imaging (DI) is commonly ordered contrary to guideline recommendations for patients with LBP. This study seeks to determine if physician characteristics are associated with ordering of DI for patients with non-specific or mechanical LBP in the ED. These characteristics include physician sex, age, experience level, location of residency training, and full-time status.

Methodology

We included all patients presenting to the ED of a Canadian tertiary care center with a diagnosis of non-specific or mechanical LBP between January 2015 and June 2018. We tracked the use of DI for physicians caring for patients presenting to the ED over this period. Simple and multivariable logistic regression analyses were performed, controlling for patient characteristics, to identify provider characteristics that were independently associated with DI use.

Results

Internationally trained physicians were less likely to order diagnostic radiographs than Canadian trained physicians (odds ratio [OR], 0.625; 95% confidence interval [CI], 0.48-0.95), while middle-aged physicians ordered more computed tomography scans (OR, 6.34; 95% CI, 1.52-26.52) compared to younger physicians; there was no significant difference between younger and older physicians.

Conclusions

Few physician characteristics were associated with any DI ordering for non-specific or mechanical LBP. The likelihood of receiving DI for non-specific or mechanical LBP may be more strongly related to unmeasured patient characteristics, settings, or logistical factors.

## Introduction

The lifetime prevalence of low back pain (LBP) for individuals in developed countries is estimated to be between 49% and 90% [1]. LBP is also one of the leading causes for emergency department (ED) visits [2]. A recent systematic review and meta-analysis of 21 studies reported that approximately 4.4% of ED visits in a busy urban center were for LBP [2]. Most LBP cases are “non-specific,” defined as without an identifiable cause [3]. Diagnostic imaging (DI) is frequently ordered as part of the investigation for LBP within the ED [4]. In 2010, Friedmann et al. [5] found that nearly one-third of patients presenting with LBP to EDs in the United States received DI.

Evidence-based guidelines recommend that DI should only be ordered for LBP presentations in the presence of one or more “red flags,” which are symptoms suggestive of a serious underlying condition [6-9]. Furthermore, they state that low back DI is indicated only when there are signs of severe, progressive neurologic deficits or symptoms that suggest an underlying condition [10]. Examples of these red flags include, but are not limited to, sudden or progressive onset of new urinary retention, fecal incontinence, saddle anesthesia, radicular leg pain often bilateral, loss of voluntary rectal sphincter contraction, severe unremitting pain, significant trauma, weight loss, fever, history of cancer or HIV, use of IV drugs or steroids, widespread neurological signs, and patient age above 50, particularly above 65 with first episode of back pain [8]. It should be noted that these “red flag” symptoms appear in many LBP clinical guidelines but individually do not necessarily indicate the need for DI. The American College of Physicians and the American Pain Society advise that patients with non-specific LBP should not be imaged as part of routine care [10].

Studies have demonstrated that physician characteristics influence physician decision-making in clinical scenarios involving pain management [11,12], thus impacting the overall delivery of health care. In particular, the influence of physician characteristics on clinical decision-making has been demonstrated as having an impact on LBP management in settings outside of the ED [13,14], and on the use of DI for LBP [15,16]. Past research has explored the association of various factors in relation to physician decision-making and use of DI for evaluation of LBP. These factors have included physician access to DI, patient behavior, and previous DI for the patient [17,18]. However, few studies have comprehensively examined physician characteristics in relation to the decision to order DI for LBP [13,14], and this is particularly underexplored in the ED setting.

To address this gap in knowledge, the purpose of this study was to determine if physician characteristics including physician sex, age, experience level, location of residency training, and full-time status were associated with ordering of DI for LBP in the ED setting.

## Materials and methods

Data Sources

We linked data from the Emergency Department Information System (EDIS) of the Charles V. Keating Emergency and Trauma Centre (QEII ED) and the Dalhousie Department of Emergency Medicine Faculty Database. EDIS was used to gather information on patient and visit characteristics. The Dalhousie Department of Emergency Medicine Faculty Database provided attending physician characteristics for all treating physicians.

Participants

Patients: We included all patients presenting to the QEII ED diagnosed with non-specific or mechanical LBP between January 1st, 2015 and June 15th, 2018 in our study population. The study site is the largest emergency care center in Atlantic Canada and is located in Halifax, Nova Scotia. Each year, there are approximately 1,400 non-specific or mechanical LBP visits to the QEII ED [4].

For this study, non-specific LBP was defined as LBP with no identifiable cause or pathology. Mechanical back pain was defined as LBP without accompanying neurological signs and symptoms. We identified eligible patients with non-specific and mechanical LBP-based ED diagnoses using ICD-9 codes.

Physicians: We included all emergency medicine physicians practicing at the QEII ED who treated an eligible patient during the reference period.

Variables of Interest

Data on sex, presenting level of pain intensity, primary care provider availability, method of arrival, time of presentation, Canadian Triage and Acuity Scale (CTAS) at presentation, and type of ED visit were extracted from the EDIS database.

Data on physician sex, experience level, age, degree presenting institution, and full-time work status were extracted from the Dalhousie Department of Emergency Medicine Faculty Database.

Certain patient characteristics may potentially confound our investigation of physician characteristics. We reviewed the literature to determine the set of patient characteristics that may impact the decision of physicians to order DI in this setting [8,10-12,18-20]. Four main constructs of specific confounding patient characteristics were considered: patient red flag characteristics, severity perception, interactions with other healthcare professionals, and time-sensitive characteristics.

Outcome

Our primary outcome variable, use of lumbar-specific DI, was obtained from the EDIS database. We coded “use of DI” or “no use of DI” for each patient encounter, in addition to use of each type of imaging (radiographs, CT, magnetic resonance imaging [MRI]).

Statistical Analysis

We describe our study population, including patient and physician characteristics using means and 95% confidence intervals (CIs) for normally distributed continuous variables, and frequencies and proportions to describe categorical variables. We describe the use of DI ordered for various physician and patient characteristics, reported as frequencies and proportions.

We investigated the relationship between patient, setting, and physician characteristics and use of DI using unadjusted and adjusted logistic regression analyses. We selected patient and setting characteristics that may confound the relationship between physician characteristics and DI use based on a prespecified framework of potentially clinically important characteristics. Logistic regression models were used to estimate the association between patient characteristics and DI use within each of our four predefined patient/setting variable constructs: red flag criteria (defined as age categories 46-55 and above in our dataset, and ED diagnoses of radiculopathy, leg radiculopathy, herniated disc, degenerative disc disease, neuralgia, leg weakness, and sciatica), LBP severity perception (arriving by ambulance, emergent CTAS score, presenting during work hours, healthcare provider suggested visit, severe pain), interactions with other healthcare professionals (arriving by ambulance, patient has a primary care provider, healthcare provider suggested visit, worker compensation), and time-sensitive characteristics (presenting during busy hours, emergent CTAS score). Variables measuring these constructs were then used as covariates in the multivariable logistic regression analysis of physician characteristics.

We used STATA 14 Version 2 (14.2) (StataCorp LLC, Texas, USA) software for all data analyses.

Ethics

This study was approved by the Nova Scotia Health Authority Research Ethics Board (ROMEO #1020036).

## Results

There were 5,213 patient visits to the ED between January 1st, 2015 and June 15th, 2018 where the patients received a diagnosis of non-specific or mechanical LBP. A total of 991 (19.01%) patients received lumbar spine DI during the visit. Radiographs accounted for 88.0% of the modality used for DI, while CT scans and MRI accounted for 11.4% and 0.6%, respectively. A total of 27 patients received DI from multiple modalities during their visits, so the sum of each modality used (1,018) and number of patients receiving DI in Table 1 (991) vary.

**Table 1:** Study population description of patients and physician interactions using proportions and percentages. DI, diagnostic imaging; CT, computed tomography; MRI, magnetic resonance imaging; PCP, primary care provider; CTAS, Canadian Triage and Acuity Scale; ED, emergency department; LBP, low back pain; HCP, healthcare professional; WCB, Workers Compensation Board

Patient characteristics	Number of patients	Patients receiving DI (%)	Patients receiving radiographs (%)	Patients receiving CT (%)	Patients receiving MRI (%)
All	5,213	991 (19.01)	896 (17.2)	116 (2.23)	6 (0.12)
Sex (Missing = 0)					
Female	2,753	561 (20.37)	518 (18.82)	55 (19.98)	1 (0.04)
Male	2,460	430 (17.56)	378 (15.27)	61 (24.80)	5 (0.20)
Presenting level of pain (scale) (Missing = 2,841)					
Mild (1-3)	179	38 (21.22)	35 (19.55)	5 (2.79)	0 (0)
Moderate (4-6)	2,009	351 (17.47)	318 (15.83)	35 (1.74)	4 (0.20)
Severe (7-10)	653	127 (19.45)	108 (16.54)	22 (3.37)	2 (0.31)
Age (years) (Missing = 0)					
16-25	763	88 (11.53)	84 (11.01)	5 (0.66)	0 (0)
26-35	1,008	131 (13.00)	116 (11.51)	15 (1.49)	2 (0.20)
36-45	928	112 (12.07)	101 (10.88)	12 (1.29)	2 (0.22)
46-55	976	183 (18.75)	156 (15.98)	30 (3.07)	2 (0.20)
56-65	684	137 (20.03)	125 (18.27)	14 (2.05)	0 (0)
66-75	461	150 (32.54)	132 (28.63)	25 (5.42)	0 (0)
76+	393	190 (48.35)	182 (46.31)	15 (3.82)	0 (0)
PCP (Missing = 0)					
Does have PCP	4,437	894 (20.15)	808 (18.21)	105 (2.37)	6 (0.14)
Does not have PCP	776	97 (12.50)	88 (11.34)	11 (1.42)	0 (0)
Method of arrival (Missing = 16)					
Independently	2,751	451 (16.39)	411 (14.94)	43 (1.56)	5 (0.18)
Relative	1,261	242 (19.19)	214 (16.97)	31 (2.46)	1 (0.08)
Ambulance	878	256 (29.16)	233 (26.54)	37 (4.21)	0 (0)
Friend	307	40 (13.03)	36 (11.72)	5 (1.53)	0 (0)
Time of presentation (Missing = 0)					
Presenting during work hours (8 am to 5 pm)	3,253	668 (20.53)	601 (18.48)	81 (2.49)	5 (0.15)
Not presenting during work hours (8 am to 5 pm)	1,960	323 (16.48)	295 (15.05)	35 (1.79)	1 (0.05)
Presenting on a weekday (Monday-Friday)	3,853	731 (18.97)	663 (17.21)	77 (19.98)	5 (0.13)
Presenting on a weekend (Saturday-Sunday)	1,360	260 (19.12)	233 (17.13)	39 (2.87)	1 (0.07)
Off-hours (every time outside 8 am to 5 pm weekdays)	2,824	504 (17.85)	457 (16.18)	62 (2.20)	2 (0.04)
Busy hours (8 am to 5 pm weekdays)	2,389	487 (20.39)	439 (18.38)	54 (2.26)	4 (0.17)
CTAS (Missing = 0)					
2 (Emergent)	498	127 (25.50)	96 (19.28)	32 (6.43)	5 (1.00)
3 (Urgent)	2,455	539 (21.96)	489 (19.92)	64 (2.61)	1 (0.04)
4 (Less urgent)	2,199	319 (14.51)	306 (13.92)	19 (0.86)	0 (0)
5 (Non-urgent)	61	6 (9.84)	5 (8.20)	1 (1.64)	0 (0)
Type of ED visit (Missing = 1)					
Emergency presentation	5,133	982 (19.13)	891 (17.36)	113 (2.20)	5 (0.10)
Other (HCP suggested visit)	79	9 (11.39)	5 (6.33)	3 (3.80)	1 (1.27)
ED diagnosis categories (main problem) (Missing = 1)					
Non-specific LBP	3,849	772 (20.06)	702 (18.24)	87 (2.26)	3 (0.08)
Mechanical LBP	1,364	219 (16.06)	194 (14.22)	29 (2.13)	3 (0.22)
Workers compensation payment (Missing = 0)					
Other payment	4,799	941 (19.61)	848 (17.67)	111 (2.31)	6 (0.13)
WCB payment	414	50 (12.08)	48 (11.59)	5 (1.21)	0 (0)
Physician characteristics	Number of physician interactions	Patients receiving DI (%)	Patients receiving radiograph (%)	Patients receiving CT (%)	Patients receiving MRI (%)
All	5,213	991 (19.01)	896 (17.2)	116 (2.23)	6 (0.12)
Sex (Missing = 0)					
Male	3,519	641 (18.22)	583 (16.57)	66 (2.16)	5 (0.14)
Female	1,694	350 (20.66)	313 (18.48)	50 (2.95)	1 (0.06)
Experience level (years worked) (Missing = 0)					
Early career (<10years)	838	158 (18.85)	139 (16.59)	20 (2.39)	1 (0.12)
Mid-career (11-25 years)	2,147	385 (17.93)	343 (15.98)	50 (2.33)	2 (0.09)
Late career (26+ years)	2,228	448 (20.11)	414 (18.58)	46 (2.06)	3 (0.13)
Degree presenting institution (residency training) (Missing = 330)					
Canadian	4,219	836 (19.81)	762 (18.06)	91 (2.16)	6 (0.14)
International	664	104 (15.67)	87 (13.10)	21 (3.16)	0 (0)
Age (years) (Missing = 0)					
26-35	740	135 (18.24)	127 (17.16)	7 (0.95)	2 (0.27)
36-45	1,797	312 (17.36)	278 (15.47)	47 (2.62)	1 (0.06)
46-55	1,132	225 (19.88)	195 (17.23)	36 (3.18)	2 (0.18)
56-65	878	170 (19.36)	157 (17.88)	16 (1.82)	0 (0)
66-75	415	107 (25.78)	101 (24.34)	6 (1.45)	1 (0.24)
76+	251	42 (17.13)	38 (15.14)	4 (0.016)	0 (0)
Young adults (<35 years)	740	135 (18.24)	127 (17.16)	7 (0.95)	2 (0.27)
Middle-aged adults (36-55 years)	2,929	537 (18.33)	473 (16.15)	83 (2.83)	3 (0.10)
Older adults (56+ years)	1,544	319 (20.66)	296 (19.17)	26 (1.68)	1 (0.06)
Full-time status (Missing = 0)					
Full-time	3,814	738 (19.35)	668 (17.51)	89 (2.33)	4 (0.10)
Part-time	1,399	253 (18.08)	228 (16.30)	27 (1.93)	2 (0.14)

Unadjusted logistic regression analysis was conducted for patient characteristic variables for use of any DI. Older age categories were progressively more likely to receive DI than the youngest patients [46-55 years (OR, 1.77; 95% CI, 1.35-2.33), 56-65 years (OR, 1.92; 95% CI, 1.44-2.57), 66-75 years (OR, 3.70; 95% CI, 2.75-4.97), above 76 years (OR, 7.18; 95% CI, 5.33-9.67)]. Those arriving by ambulance were also more likely to receive DI (OR, 2.10; 95% CI, 1.76-2.51). We found the following patient characteristic variables to have a reduced likelihood of receiving DI: patient does not have a primary care provider (OR, 0.566; 95% CI, 0.45-0.71), CTAS scores of 4 (OR, 0.496; 95% CI, 0.39-0.62), and 5 (OR, 0.319; 95% CI, 0.13-0.76), healthcare provider suggested visit (OR, 0.297; 95% CI, 0.11-0.82), and workers compensation payment (OR, 0.560; 95% CI, 0.42-0.76). Results for associations of other patient characteristics with any DI are outlined in Table 2.

**Table 2:** Simple logistic regression analysis results for patient and physician characteristics for any DI. DI, diagnostic imaging; OR, odds ratio; PCP, primary care provider; CTAS, Canadian Triage and Acuity Scale; ED, emergency department; HCP, healthcare professional; WCB, Workers Compensation Board

Patient characteristics	DI-unadjusted OR
Sex (Missing = 0)	
Female	Reference
Male	0.827 (0.72-0.95) p = 0.008
Presenting level of pain (Scale) (Missing = 2,841)	
Mild (1-3)	Reference
Moderate (4-6)	0.785 (0.54-1.14) p = 0.209
Severe (7-10)	0.896 (0.60-1.35) p = 0.597
Age (years) (Missing = 0)	
16-25	Reference
26-35	1.14 (0.86-1.53) p = 0.355
36-45	1.05 (0.78-1.42) p = 0.734
46-55	1.77 (1.35-2.33) p < 0.001
56-65	1.92 (1.44-2.57) p < 0.001
66-75	3.70 (2.75-4.97) p < 0.001
76+	7.18 (5.33-9.67) p < 0.001
PCP (Missing = 0)	
Does have PCP	Reference
Does not have PCP	0.566 (0.45-0.71) p = <0.001
Method of arrival (Missing = 16)	
Independently	Reference
Relative	1.21 (1.02-1.44) p = 0.030
Ambulance	2.10 (1.76-2.51) p < 0.001
Friend	0.764 (0.54-1.08) p = 0.129
Time of presentation (Missing = 0)	
Presenting during work hours (8 am to 5 pm)	Reference
Not presenting during work hours (8 am to 5 pm)	0.764 (0.66-0.88) p < 0.001
Presenting on a weekday (Monday-Friday)	Reference
Presenting on a weekend (Saturday-Sunday)	1.01 (0.86-1.18) p = 0.906
Off-hours (every time outside 8 am to 5 pm weekdays)	Reference
Busy hours (8 am to 5 pm weekdays)	1.18 (1.03-1.35) p = 0.02
CTAS (Missing = 0)	
2 (Emergent)	Reference
3 (Urgent)	0.822 (0.66-1.03) p = 0.085
4 (Less urgent)	0.496 (0.39-0.62) p < 0.001
5 (Non-urgent)	0.319 (0.13-0.76) p = 0.01
Type of ED visit (Missing = 1)	
Emergency Presentation	Reference
Other (HCP suggested visit)	0.297 (0.11-0.82) p = 0.019
Workers compensation (Payment Missing = 0)	
Other payment	Reference
WCB payment	0.560 (0.42-0.76) p < 0.001
Physician characteristics	
Sex (Missing = 0)	
Male	Reference
Female	1.17 (1.01-1.35) p = 0.035
Experience level (years worked) (Missing = 0)	
Early career (<10 years)	Reference
Mid-career (11-25 years)	0.940 (0.77-1.15) p = 0.557
Late career (26+ years)	1.08 (0.89-1.33) p = 0.437
Degree presenting institution (residency training) (Missing = 330)	
Canadian	Reference
International	0.752 (0.60-0.94) p = 0.012
Age (years) (Missing = 0)	
26-35	Reference
36-45	0.942 (0.75-1.18) p = 0.597
46-55	1.11 (0.88-1.41) p = 0.381
56-65	1.08 (0.84-1.38) p = 0.566
66-75	1.56 (1.17-2.08) p = 0.003
76+	0.901 (0.62-1.32) p = 0.589
Young adults (<35 years)	Reference
Middle-aged adults (36-55 years)	1.01 (0.82-1.24) p = 0.955
Older adults (56+ years)	1.17 (0.93-1.46) p = 0.176
Full-time status (Missing = 0)	
Full-time	Reference
Part-time	0.920 (0.79-1.08) p = 0.302

For diagnostic radiographs specifically, further investigation with unadjusted logistic regression revealed older patients to be progressively more likely to receive diagnostic radiographs than younger patients [46-55 years (OR, 1.54; 95% CI, 1.16-2.05), 56-65 years (OR, 1.81; 95% CI, 1.34-2.44), 66-75 years (OR, 3.24; 95% CI, 2.39-4.39), above 76 years (OR, 6.97; 95% CI, 5.16-9.42)]. Similarly, those arriving by ambulance were also more likely to receive diagnostic radiographs (OR, 2.06; 95% CI, 1.71-2.47). We found the following patient characteristic variables to have a reduced likelihood of receiving diagnostic radiographs: patient does not have primary care provider (OR, 0.574; 95% CI, 0.45-0.73), CTAS score of 5 (OR, 0.374; 95% CI, 0.15-0.96), healthcare provider suggested visit (OR, 0.334; 95% CI, 0.12-0.92), and workers compensation payment (OR, 0.610; 95% CI, 0.45-0.83). Results for associations of other patient characteristics with diagnostic radiographs are outlined in Table 3.

For CT, unadjusted logistic regression revealed the following to be patient variables associated with CT; ages 46-55 years (OR, 4.81; 95% CI, 1.86-12.45), 56-65 years (OR, 3.17; 95% CI, 1.13-8.84), 66-75 years (OR, 8.69; 95% CI, 3.30-22.87), above 76 years (OR, 6.01; 95% CI, 2.17-16.68), arriving by ambulance (OR, 2.77; 95% CI, 1.77-4.33), and CTAS score of 3 (OR, 0.390; 95% CI, 0.25-0.60) and 4 (OR, 0.127; 95% CI, 0.07-0.23). Results for associations of other patient characteristics with CT are outlined in Table 3.

**Table 3:** Simple logistic regression analysis results for patient and physician characteristics for diagnostic radiographs and CT. OR, odds ratio; CT, computed tomography; PCP, primary care provider; CTAS, Canadian Triage and Acuity Scale; ED, emergency department; HCP, healthcare professional; WCB, Workers Compensation Board

Patient characteristics	Radiographs-unadjusted OR	CT-unadjusted OR
All	N/A	N/A
Sex (Missing = 0)		
Female	Reference	Reference
Male	0.784 (0.68-0.91) p = 0.001	1.25 (0.86-1.80) p = 0.240
Presenting level of pain (Scale) (Missing = 2,841)		
Mild (1-3)	Reference	Reference
Moderate (4-6)	0.774 (0.52-1.14) p = 0.195	0.617 (0.24-1.60) p = 0.319
Severe (7-10)	0.815 (0.53-1.24) p = 0.344	1.21 (0.45-3.25) p = 0.701
Age (years) (Missing = 0)		
16-25	Reference	Reference
26-35	1.05 (0.78-1.42) p = 0.743	2.29 (0.83-6.33) p = 0.110
36-45	0.987 (0.726-1.34) p = 0.934	1.99 (0.70-5.66) p = 0.199
46-55	1.54 (1.16-2.05) p = 0.003	4.81 (1.86-12.45) p = 0.001
56-65	1.81 (1.34-2.44) p < 0.001	3.17 (1.13-8.84) p = 0.028
66-75	3.24 (2.39-4.39) p < 0.001	8.69 (3.30-22.87) p < 0.001
76+	6.97 (5.16-9.42) p < 0.001	6.01 (2.17-16.68) p = 0.001
PCP (Missing = 0)		
Does have PCP	Reference	Reference
Does not have PCP	0.574 (0.45-0.73) p = <0.001	0.593 (0.32-1.11) p = 0.102
Method of arrival (Missing = 16)		
Independently	Reference	Reference
Relative	1.16 (0.97-1.39) p = 0.100	1.59 (1.0-2.53) p = 0.052
Ambulance	2.06 (1.71-2.47) p < 0.001	2.77 (1.77-4.33) p < 0.001
Friend	0.756 (0.53-1.09) p = 0.132	1.04 (0.41-2.65) p = 0.903
Time of presentation (Missing = 0)		
Presenting during work hours (8 am to 5 pm)	Reference	Reference
Not presenting during work hours (8 am to 5 pm)	0.782 (0.67-0.91) p < 0.001	0.712 (0.48-1.06) p = 0.096
Presenting on a weekday (Monday-Friday)	Reference	Reference
Presenting on a weekend (Saturday-Sunday)	0.995 (0.84-1.17) p = 0.950	1.45 (0.98-2.14) p = 0.063
Off-hours (every time outside 8 am to 5 pm weekdays)	Reference	Reference
Busy hours (8 am to 5 pm weekdays)	1.17 (1.01-1.35) p = 0.037	1.03 (0.71-1.49) p = 0.874
CTAS (Missing = 0)		
2 (Emergent)	Reference	Reference
3 (Urgent)	1.04 (0.82-1.33) p = 0.743	0.390 (0.25-0.60) p < 0.001
4 (Less urgent)	0.677 (0.53-0.87) p = 0.003	0.127 (0.07-0.23) p < 0.001
5 (Non-urgent)	0.374 (0.15-0.96) p = 0.041	0.243 (0.03-1.81) p = 0.167
Type of ED visit (Missing = 1)		
Emergency presentation	Reference	Reference
Other (HCP suggested visit)	0.334 (0.12-0.92) p = 0.035	1
Workers compensation payment (Missing = 0)		
Other payment	Reference	Reference
WCB payment	0.610 (0.448-0.833) p = 0.002	0.520 (0.21-1.27) p = 0.151
Physician characteristics	Radiographs-unadjusted OR	CT-Unadjusted OR
Sex (Missing = 0)		
Male	Reference	Reference
Female	1.14 (0.98-1.33) p = 0.087	1.59 (1.10-2.31) p = 0.014
Experience level (years worked) (Missing = 0)		
Early career (<10 years)	Reference	Reference
Mid-career (11-25 years)	0.956 (0.77-1.19) p = 0.683	0.975 (0.58-1.65) p = 0.925
Late career (26+ years)	1.15 (0.93-1.42) p = 0.201	0.862 (0.51-1.47) p = 0.584
Degree presenting institution (residency training) (Missing = 330)		
Canadian	Reference	Reference
International	0.684 (0.54-0.87) p = 0.002	1.48 (0.92-2.40) p = 0.110
Age (years) (Missing = 0)		
26-35	Reference	Reference
36-45	0.88 (0.702-1.11) p = 0.290	2.81 (1.27-6.25) p = 0.011
46-55	1.00 (0.786-1.28) p = 0.971	3.34 (1.52-7.77) p = 0.003
56-65	1.05 (0.81-1.36) p = 0.705	1.94 (0.80-4.75) p = 0.145
66-75	1.55 (1.16-2.08) p = 0.003	1.54 (0.513-4.60) p = 0.443
76+	0.861 (0.580-1.28) p = 0.458	1.70 (0.492-5.84) p = 0.403
Young adults (<35 years)	Reference	Reference
Middle-aged adults (36-55 years)	0.930 (0.75-1.15) p = 0.506	3.05 (1.41-6.63) p = 0.005
Older adults (56+ years)	1.15 (0.91-1.44) p = 0.248	1.79 (0.77-4.15) p = 0.172
Full-time status (Missing = 0)		
Full-time	Reference	Reference
Part-time	0.917 (0.78-1.08) p = 0.302	0.823 (0.53-1.27) p = 0.382

Multivariable logistic regression analysis to examine physician characteristics associated with the use of DI, while controlling for patient and setting characteristics, found that internationally trained physicians were significantly less likely to order diagnostic radiographs (OR, 0.692; 95% CI, 0.49-0.97) in comparison to their Canadian trained colleagues. Middle-aged physicians (ages 36-55) were significantly more likely to order CT scans (OR, 6.29; 95% CI, 1.50-26.34) in comparison to younger physicians (OR, 3.65; 95% CI, 0.81-16.43), while no significant difference was found between younger and older physicians (Table 4).

**Table 4:** Multivariate logistic regression: provider Characteristics adjusted for patient characteristics in four domains: patient red flag characteristics, LBP severity perception, interactions with other healthcare professionals, and time-sensitive characteristics. DI, diagnostic imaging; OR, odds ratio; CT, computed tomography

Characteristics	DI-adjusted OR	Radiographs-adjusted OR	CT-adjusted OR
Physician sex			
Male	Reference	Reference	Reference
Female	0.955 (0.77-1.18) p = 0.671	0.976 (0.78-1.22) p = 0.828	1.07 (0.62-1.86) p = 0.808
Physician age			
Young adults (26-35 years)	Reference	Reference	Reference
Middle-aged adults (36-55 years)	0.937 (0.70-1.26) p = 0.665	0.852 (0.63-1.15) p = 0.297	6.29 (1.50-26.34) p = 0.012
Older adults (56+ years)	1.11 (0.81-1.53) p = 0.506	1.07 (0.77-1.48) p = 0.690	3.65 (0.81-16.43) p = 0.091
Physician experience level			
Early career (0-10 years)	Reference	Reference	Reference
Mid-career (11-25 years)	0.850 (0.64-1.13) p = 0.266	0.857 (0.64-1.16) p = 0.312	1.13 (0.53-2.40) p = 0.799
Late career (26+ years)	0.916 (0.69-1.21) p = 0.540	0.931 (0.69-1.25) p = 0.635	0.984 (0.46-2.12) p = 0.967
Location of residency program			
Canadian	Reference	Reference	Reference
International	0.767 (0.56-1.05) p = 0.099	0.692 (0.49-0.97) p = 0.033	1.35 (0.67-2.73) p = 0.396
Physician full-time status			
Full-time	Reference	Reference	Reference
Part-time	1.06 (0.84-1.32) p = 0.634	1.07 (0.85-1.36) p = 0.555	0.724 (0.38-1.39) p = 0.331

## Discussion

This study confirmed that DI is often ordered for patients who present to the ED and are ultimately diagnosed with non-specific or mechanical LBP, with 19% of the patients receiving imaging. Other studies have found that this commonly occurs without high pre-test probability of a positive finding, unnecessarily exposing patients to the harms of radiation [8,9].

Our analysis found that patients were significantly less likely to receive diagnostic radiographs from physicians who were internationally trained compared to those from Canadian programs. Over-utilization of DI is a well-documented occurrence in developed countries [21]. In developing countries, lack of access to DI equipment remains an issue and may contribute to a culture within medical schools discouraging DI for screening purposes. However, we were unable to determine if internationally trained emergency physicians within this study came from medical schools of developing countries. Furthermore, it should be noted that, although the only significant finding pertaining to internationally trained physicians was specific to radiographs, a non-significant finding of reduced OR to order any type of DI was also present for internationally trained physicians. A similar finding was reported in a study which found primary care physicians who were trained outside of the United States were less likely to order CT scans or MRI for acute LBP [19].

Studies have indicated that CT use in the ED has grown dramatically over the last two decades [22,23]. Another important finding in our study is that middle-aged physicians were significantly more likely to order CT scans for LBP in the ED in comparison to their colleagues who are younger. Furthermore, we found that older physicians were more likely to order CT scans for patients but to a lesser extent than middle-aged physicians; however, this result was not statistically significant. This observation points to a potential trend where younger physicians are less likely to order CT scans. A similar trend was demonstrated among physicians in another Canadian study, where older physicians were more likely to order radiographs in comparison to their younger colleagues [24]. A possibility as to why younger physicians less frequently order CT imaging is due to the awareness of over-ordering diagnostic tests that has been better implemented into the curriculum of younger physicians [24]. CT scan reduction is of particular importance because of the large radiation exposure and the greater likelihood of potential future health concerns for patients [25].

We were surprised to find that none of the physician characteristics investigated were associated with the use of any DI overall (radiographs, CT, and MRI). However, several physician and patient characteristics were found to be associated with the ordering of specific DI modalities in unadjusted analyses. We interpret these results cautiously as confounding variables were not taken into consideration for these analyses and alternative explanations can be given for many of these results through clinical reasoning. For example, it was shown that arriving by ambulance resulted in twice the likelihood (p < 0.001) that patients received any DI overall; however, patient acuity could easily confound this relationship.

A larger-scale, multi-site study examining the variation in DI ordering habits for physicians with various characteristics and more completely controlling for patient and setting characteristics would be beneficial. Furthermore, a study investigating appropriate resource utilization among various ED physician age groups could give insights into reasons for DI discrepancies. This study suggests that tailoring clinical solutions to reduce DI for LBP toward physicians with certain characteristics would be largely unhelpful. However, a benefit may be seen if middle-aged physicians were educated on current best practices for ordering CT scans for LBP. Even so, evidence suggests that available interventions to reduce unnecessary LBP DI in the ED have so far proven to be largely ineffective [26]; a trend that is common in the ED for clinical decision-making tools related to DI [27,28]. This signifies the need for additional research toward the development and, more importantly, implementation of such interventions.

Limitations

This study was subject to limitations. First, two key variables identified in the preliminary literature review of patient characteristics were not available within the available databases, namely, patient ethnicity and LBP duration. Further research would be required to determine the impact of physician and patient ethnicity and the duration of LBP complaint within the ED context. Second, this retrospective study was conducted using data from a small geographical region whose medical culture may not be generalizable to other regions. For example, one study found that patients experiencing LBP are 1.5 times more likely to receive radiographic imaging in a metropolitan ED in comparison to a rural setting [29]. Furthermore, one study demonstrated site-specific differences of four health centers within the same city [24]. Finally, it was not possible to determine whether it was the attending physician or a medical resident who ordered the DI. One study reported that residents were 2.5 to 4.5 more likely to order DI in this context in comparison to ED physicians [26]. The QEII Health Sciences Centre is an established teaching hospital, and therefore, many residents are responsible for LBP management within the ED. However, many physicians validate the choice of DI when residents order it.

## Conclusions

This study demonstrated a higher association of ordered CT and radiograph studies for patients whose treating physician were middle-aged or trained in Canada, respectively. However, no physician characteristic was associated with the use of any DI overall. This study suggests that the odds of receiving DI for non-specific or mechanical LBP may be more strongly associated with patient, environmental, or organizational factors than physician characteristics alone. Future research on this topic should focus on expanding physician variables for analysis.
